# Evaporated C60 as fast anode for thin-film solid-state batteries

**DOI:** 10.1080/14686996.2026.2726180

**Published:** 2026-09-21

**Authors:** Jędrzej Morzy, Niels Uythoven, Yaroslav E. Romanyuk

**Affiliations:** Laboratory for Thin Films and Photovoltaics, Empa – Swiss Federal Laboratories for Materials Science and Technology, Dübendorf, Switzerland

**Keywords:** Batteries, thin-film, electrochemistry, anode, C60, fullerene, solid-state

## Abstract

Thin-film solid-state batteries require anode materials that combine fast lithium storage, cycling stability, and compatibility with scalable vacuum processing to enable novel applications. Here, we demonstrate thermally evaporated C_60_ based films as high-rate anodes in planar LiPON-based thin-film solid-state half-cells. Structural characterization by X-ray diffraction and transmission electron microscopy shows that the as-deposited films lack long-range fullerite crystallinity and are best described as structurally disordered C_60_-derived layers. Electrochemically, the 200 nm films deliver reversible multistep lithiation with capacities up to ~560 mAh g^−1^, exceeding the nominal Li_12_C_60_ composition, and retain substantial capacity at extreme C-rates. The cells provide ~410 mAh g^−1^ at 1 mA cm^−2^ and ~220 mAh g^−1^ at 12 mA cm^−2^, corresponding to real rates of approximately 75 C and 1600 C, respectively. Long-term cycling is highly stable, with capacities above 400 mAh g^−1^ at 75 C maintained over more than 7000 cycles and only minor impedance growth. Impedance analysis indicates that, at the highest current densities, the cell polarization is heavily influenced by the LiPON electrolyte with C_60_ not limiting the cell performance. These results identify evaporated C_60_ as a promising Li-host anode for thin-film solid-state batteries.

## IMPACT STATEMENT

Evaporated, disordered C_60_ thin films are fast, stable, vacuum-processable anodes for solid-state microbatteries, sustaining exceptional current densities and thousands of cycles while repurposing optoelectronic molecular materials for energy storage.

## GRAPHICAL ABSTRACT

**Figure:**
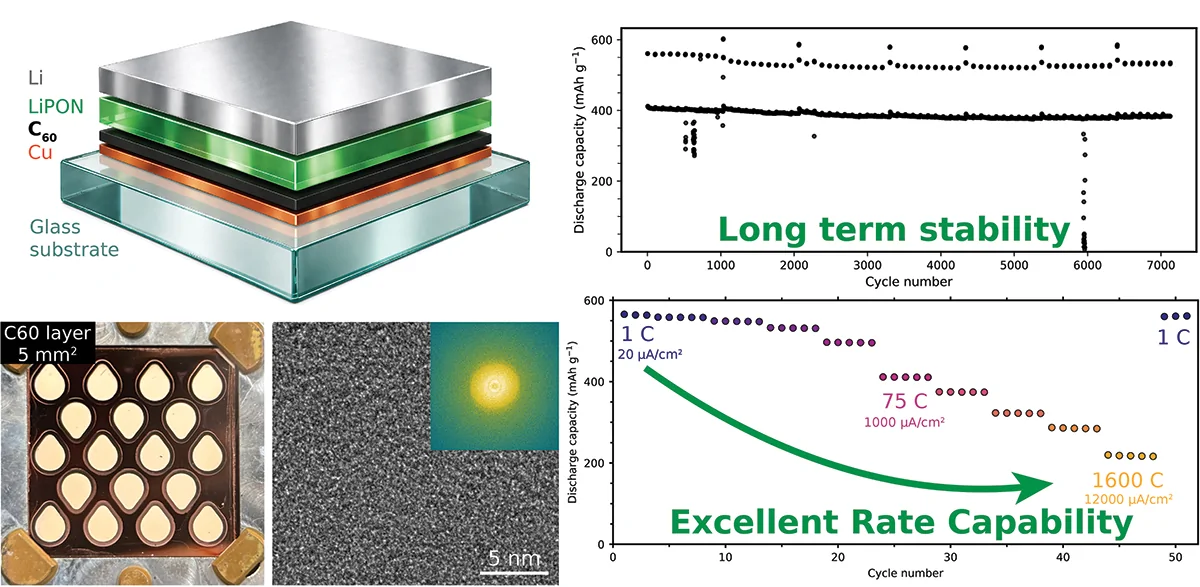

## Introduction

Thin-film solid-state batteries (TFSSBs) are an architecture with attractive properties: they are compatible with vacuum processing, the characteristic transport lengths are short (on the order of single µm), and the layered geometry provides relatively easy access to well-defined, buried interfaces [1–3]. This has made TFSSBs relevant not only for integrated power sources in microsystems and IoT devices, but also as model systems for studying electrochemical processes without the microstructural and chemical complexity of porous composite electrodes and additives [1–7].

In TFSSBs, the anode remains a critical component. Metallic Li is the most common anode material choice in LiPON-based thin-film cells because it provides the highest possible anode capacity, lowest possible potential, and supports relatively fast cycling when the interface remains stable [1,2,8–11]. Classical thin-film Li/LiPON/LiCoO_2_ cells were reported to retain more than 50% of their maximum capacity at 5 mA cm^−2^ [1], and recent thin-film seed layer work shows critical current densities for Li plating through LiPON of about 8 mA cm^−2^ with thin carbon interlayers, as relevant operating limits for Li-based or anode-free thin-film designs [6,12]. Importantly, ‘C-rate’ is not a suitable metric to judge the performance of anode layers, electrolytes or thin-film batteries as it heavily depends on the absolute capacity values. In this work, (areal) current densities will be preferred as the main comparison point. The practical limitations of Li metal are also well established. Its high reactivity imposes strict control of atmosphere during handling and processing, and its low melting point, pronounced creep, and low tensile strength complicate reproducible fabrication of thin foils and ultrathin layers [13–15]. These issues are not limited to laboratory practice; they are directly relevant to scale-up and manufacturing. In addition, Li plating and stripping result in significant volume change (assuming no initial Li reservoir, technically infinite in terms of the % change). For comparison, 0.2 mAh cm^−2^ of capacity corresponds to about 1 µm of dense Li plated and stripped on every cycle (assuming 2062 mAh/cm^3^, 0.534 g/cm^3^, 3862 mAh/g). A host-type anode can help alleviate this volume change. Several host anodes for TFSSBs have already been explored. Thermally evaporated Si is the best-known example and can deliver capacities above 2000 mAh g^−1^ [7,16–18], but such performance is coupled to large structural changes limiting lifetime and C-rate. Other thin-film anodes, including alloy anodes (e.g. Bi), nitride and oxynitride films, were also tested but generally with not as attractive performance compared to pure Li [1,19–21].

Carbonaceous materials are well known for their ability to store ions as anodes with graphite being the most prevalent example for Li [22–24]. C_60_ is molecular semiconductor that is well-established in vacuum-deposited thin-film optoelectronics, particularly photovoltaics [25–27]. C_60_ and C_70_ were evaluated for lithium batteries shortly after their discovery: Seger et al. (1991) reported that films of both materials could be reduced electrochemically in propylene carbonate/LiClO_4_ [28]. Critically, they found that reduced fullerene species are soluble in the electrolyte tested, which hindered their use in practical liquid-electrolyte batteries. Chabre et al. subsequently demonstrated electrochemical lithiation of C_60_ in solid-state cells with the maximum lithiation state of Li_12_C_60_ [29]. First-principles work by Gorelik et al. calculated the rate-limiting Li^+^ diffusion barrier in crystalline C_60_ to be ~0.34 eV, highlighting decent Li transport properties [30].

More recently, C_60_ nanoparticles were reported to exhibit high and stable (for 50 cycles) Li storage capacities of 786 mAh/g at 0.1 A/g current densities in Li-ion batteries, and >350 mAh/g at a higher current density of 5 A/g for 1000 cycles [31]. Other works also show the use of crystalline C_60_ nanorods as anode material, which provide 603 mAh/g at 2 A/g for 2000 cycles [32] as well as fullerenes that were hydrogenated or functionalized with carboxyl groups, also achieving capacities higher than for the abovementioned Li_12_C_60_ phase [33,34].

In this work, we present thermally evaporated C_60_ as a fast anode host material for TFSSBs. To the best of our knowledge, this is the first report of vacuum processed C_60_-based anode materials. Using X-ray diffraction (XRD), transmission electron microscopy (TEM), and electrochemical characterisation we demonstrate the disordered nature of the films, and the resulting reversible electrochemical behaviour with excellent capacity (>500 mAh/g) and rate performance (up to 1600 C).

## Results

**Figure 1.**
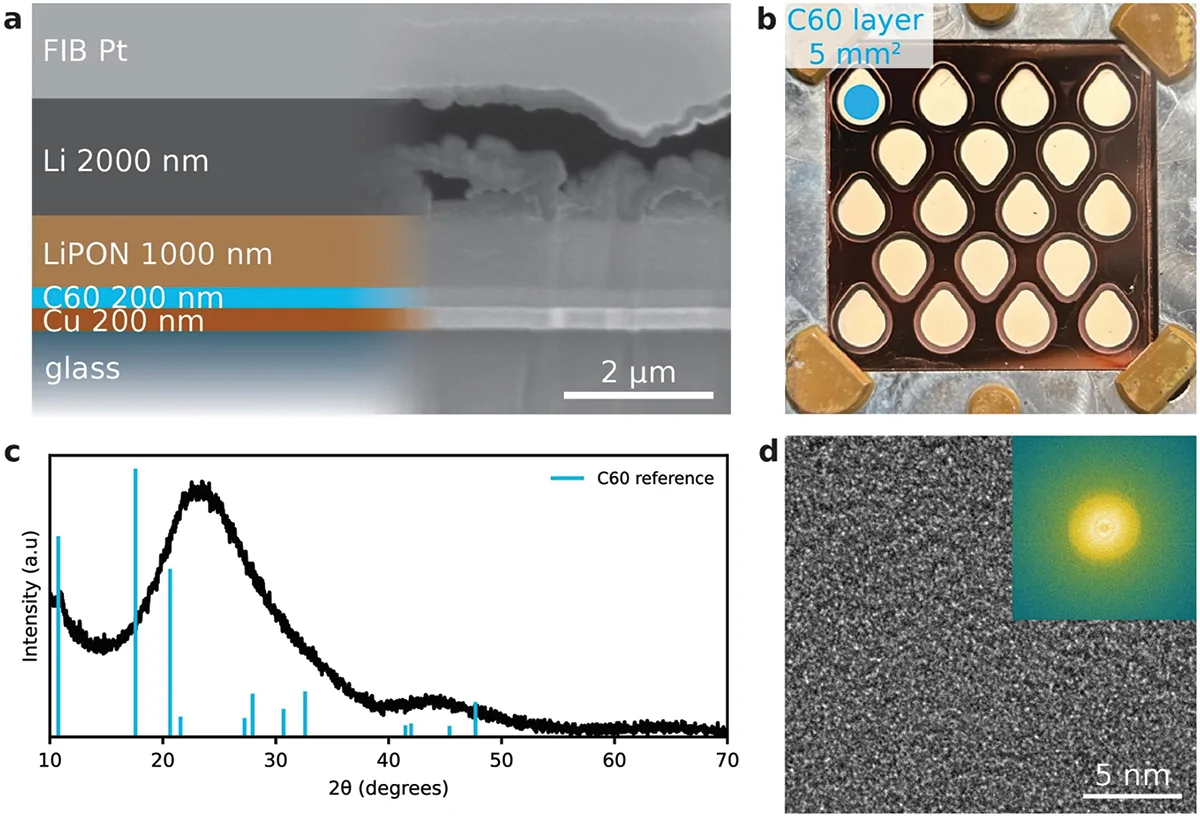
a) schematic layer structure of the half-cell and a FIB-SEM cross-sectional image (secondary electrons); b) photograph of the top, patterned surface of the sample. The lateral size of the active material layer is 5 mm^2^, covered by LiPON electrolyte (tear-drop shape, black) and Li metal counter electrode (bright gold, inset in LiPON pattern); c) XRD diffractogram of the C60 layer and reference peaks of crystalline C_60_ [35]; d) HR-TEM micrograph and corresponding FFT (inset) of the C_60_ layer.

Planar thin-film solid-state half-cells were fabricated with the following stack structure: glass/Cu current collector (200 nm)/C_60_ electrode (200 nm)/LiPON electrolyte (1000 nm)/Li counter electrode and current collector (2000 nm). This layer structure and a focused ion beam – electron microscope (FIB-SEM) cross-section are shown in Figure 1(a), while the top-view optical image of the sample geometry is shown in Figure 1(b). The masking sequence produced a well-defined active area (5 mm^2^) in which the evaporated C60 island is covered by a larger, tear-drop‑shaped LiPON region, followed by a smaller, offset Li top electrode. The cross-sectional FIB-SEM image in Figure 1(a) confirms the intended multilayer half-cell architecture and shows smooth, continuous Cu, C_60_, and LiPON layers, whereas the Li top layer appears rough and porous, due to air exposure during the transfer to the FIB-SEM.

The structural state of the active layer is investigated by X-ray diffraction (XRD) and transmission electron microscopy (TEM) measurements. The X-ray diffractogram in Figure 1 clearly shows the lack of reflections expected for crystalline fullerite C_60_. Instead, it is dominated by a broad diffuse feature centered at 2θ ≈ 23°. The HRTEM image and its fast Fourier transform (FFT) in Figure 1 support this finding. Clear lattice fringes are absent, and the FFT only contains a diffuse ring. The film was deposited from a C_60_ source, and we do not expect any C60 molecule decomposition within the process temperatures used [36,37]. XRD and TEM do not show crystalline fullerite order. Therefore, the layer is a form of a structurally disordered C_60_ film that should maintain the C_60_ molecule building block. Investigation of the molecular structure of the layer is beyond the scope of this work. For the sake of brevity, we will refer to it as the C_60_ film. The disordered nature of the films suggests that cheaper, more disordered precursors might be capable of delivering similar performance while lowering the substantial raw material cost.

**Figure 2.**
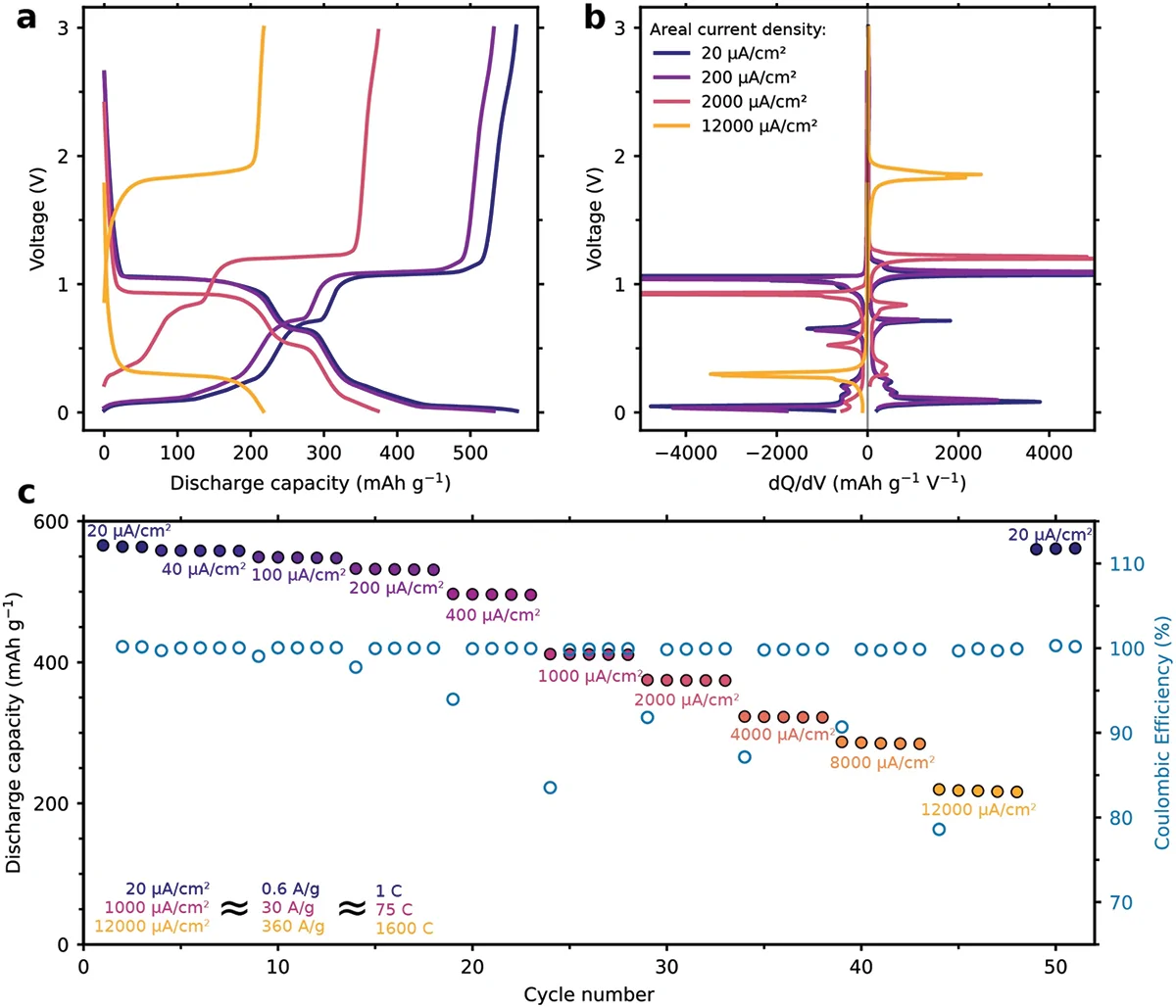
a) Voltage-capacity curves of C_60_ at selected current densities for galvanostatic cycling between 0.005 and 3 V; b) Differential capacity plots for these curves; c) C-rate test results. For the details on how the areal current density and capacity are converted to gravimetric parameters and C-rate, please see the methods section.

The C60 films were then tested for their ability to store Li as an anode in TFSSB architecture. First, the results of galvanostatic cycling of half-cells at various C-rates are shown in Figure 2(a-c). The voltage profiles in Figure 2(a) and their dQ/dV counterparts (Figure 2(b)) show that the C_60_ film is electrochemically active over a broad potential window and undergoes reversible lithiation and delithiation. The voltage curves contain distinct plateaus or quasi-plateaus, with small voltage hysteresis between charge/discharge, showing high energy efficiency of >80% at low current densities. Several voltage plateaus at 1.05 V, 0.7 V, 0.2 V, and 0.05 V can be distinguished in Figure 2(a,b), suggesting that lithium uptake does not proceed through a single uniform process. The observed plateaus are also in line with previous literature [31,34]. As the current density is raised, the hysteresis between lithiation and delithiation widens as shown in Figure 2(a), which is consistent with increasing polarization. The first cycle coulombic efficiency is measured to be 99.2% (at 1 C rate, defined as first lithiation capacity divided by the capacity of the following delithiation), indicating high reversibility of the initial lithiation and low Li inventory loss.

The same behaviour is visible more clearly in the differential-capacity plots in Figure 2(b). Several redox features can be resolved at low current density, and their positions correspond to the abovementioned plateaus. With increasing current density, the plateaus in Figure 2(a) become less ‘flat’, and the derivative capacity peaks in Figure 2(b) broaden, shift and merge with the increasing polarization overpotentials. At the highest current densities of 12,000 µA/cm^2^, corresponding to a C-rate of about 1600 C, the overpotentials reach up to 0.7 V, limiting the available (de)lithiation plateaus in the voltage window and therefore, capacity.

The rate performance is summarised in Figure 2. The C_60_ film delivers about 560 mAh g^−1^ at 20 µA cm^−2^ (corresponding to C-rate of 1C). Ideal formation of Li_12_C_60_ corresponds to ~446 mAh g^−1^ [29]. However, higher lithiation capacities have been previously demonstrated for modified C_60_-based materials, which is in line with the results of structural characterisation that demonstrate disordered nature of the films (Figure 1) [31,33,34]. The exact mechanism of Li storage in disordered/modified fullerene-based materials is still debated and beyond the scope of this work. At higher current densities, the samples provide 410 mAh g^−1^ at 1000 µA cm^−2^ (75 C), and about 220 mAh g^−1^ at 12,000 µA cm^−2^ (1600 C). The capacity decreases only slightly from 20 to 200 µA cm^−2^, and remains substantial even at the extreme current densities of 12 mA cm^−2^ (1600 C for this C_60_ thickness). Importantly, the return to 20 µA cm^−2^ (1C) cycling after the extreme C-rate test shows excellent recovery of capacity, highlighting that the film is not degraded by the high current density cycling. Even when considering the natural advantage of thin-film for fast kinetics and high C-rates, the resulting capacities at high C-rates are excellent. In the present evaporated films, the absence of long-range crystalline order may further remove crystallographic bottlenecks, grain-boundary limitations, or phase-boundary constraints associated with crystalline fullerite. At 12 mA/cm^2^, the samples are operating in a regime where the LiPON electrolyte and Li metal counter electrode themselves are likely the limiting factor as highlighted by our previous work on seed layers for Li-metal plating [6]. Therefore, the C_60_ anodes are likely to surpass the rate performance of pure Li metal anodes at extreme C-rates. This is, however, outside the scope of the current work, as it requires development of new, less resistive electrolyte and a suitable counter electrode (ideally cathode) that can sustain high current densities.

**Figure 3.**
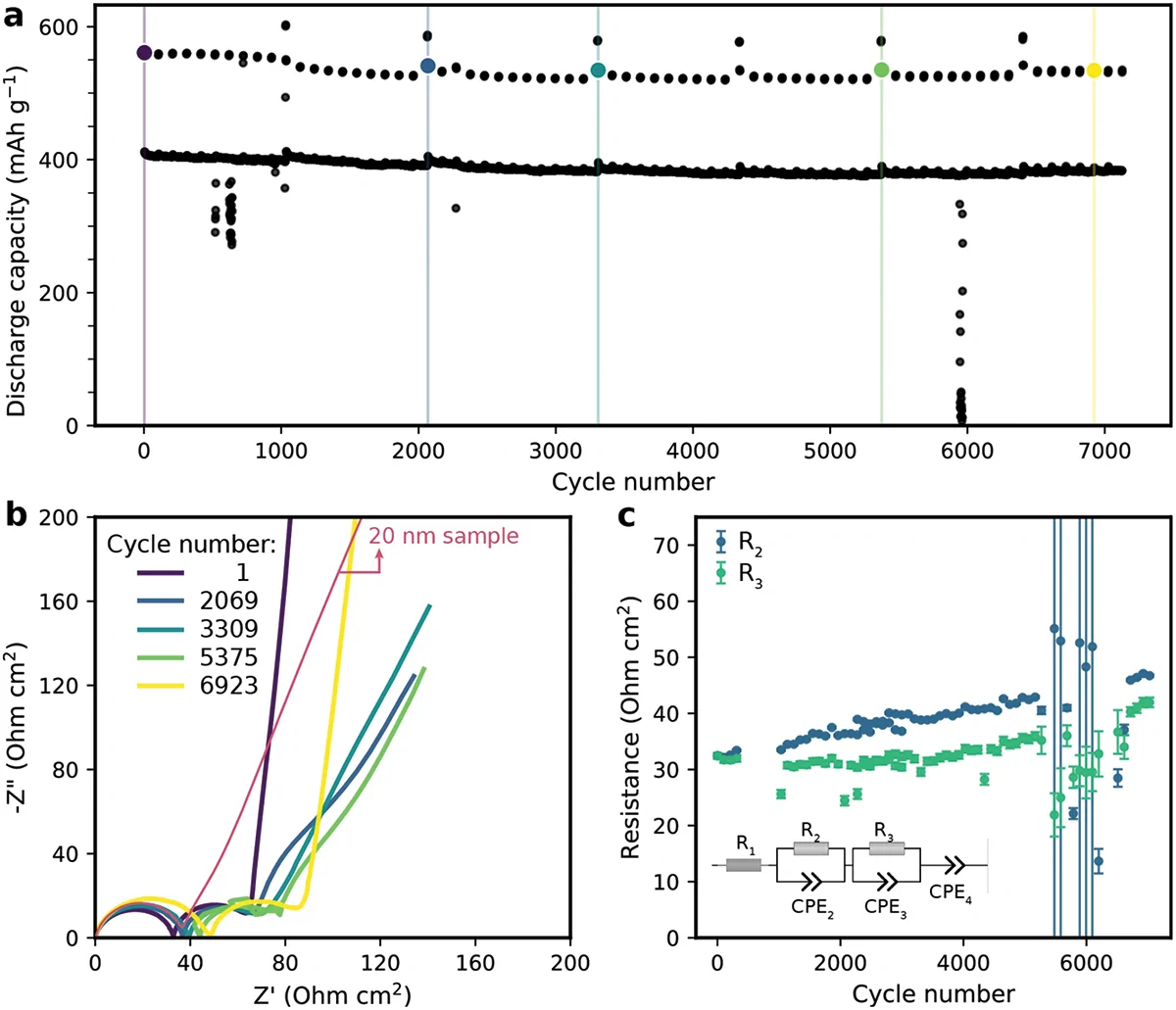
a) Long-term cycling behaviour for cycling. The cycling protocol was: 3x cycles between 0.005 and 3 V at 20 µA/cm2 (1 C, 0.6 A/g), potentiostatic EIS in the delithiated state, followed by 100x cycles with the same voltage window at 1 mA/cm2 (real rate: 75 C, 30 A/g), repeated multiple times; b) Selected EIS spectra highlighting the stability of the cell; red line corresponds to the 20 nm C_60_-based half-cell to compare the C_60_ contribution to impedance. c) Fitted resistances R_2_ and R_3_ of the EIS spectra. The error bars are the standard deviation of the fitted resistances. Inset is the equivalent circuit used.

Figure 3(a-c) shows the results of a long-term cycling experiment. The cycling protocol for the data shown in Figure 3(a) consisted of three cycles at 20 µA cm^−2^ (1C), followed by potentiostatic EIS in the delithiated state, and then 100 cycles at 1 mA cm^−2^ (75 C). This protocol was then repeated. The high rate of testing has been chosen to complete many thousands of cycles in a reasonable timeframe. The cycling behaviour is very stable throughout the >7000 cycles shown here (the cell is still running at the time of writing). The C_60_ provides gravimetric capacities of >500 mAh/g at 1C and >400 mAh/g at 75 C throughout the test. The minor fluctuations of capacity are caused by the changing temperature of the glovebox in which the testing is done. The corresponding electrochemical impedance (EIS) spectra in Figure 3(b) show minimal changes during cycling. The small changes to the spectrum are caused by fluctuating temperature during the measurements. Figure 3 shows the results of equivalent circuit model fitting with the model shown inset. It is composed of an R_1_ contribution, which represents the series resistance of cables and current collectors, two R-CPE components, which correspond to LiPON electrolyte (high frequency, R_2_, CPE_2_ see also our previous work [38]) and the bulk C60 resistance (medium frequency, R_3_, CPE_3_) followed by a polarisation tail (CPE_4_). This is supported by comparing with an equivalent sample, where the thickness of C60 was reduced to 20 nm, to rule out any contributions from interfacial and charge-transfer effects. In that cell, there is no significant contribution in the mid-frequency regime, the second semicircle (R_3_-CPE_3_) essentially disappears (Figure 3(b), red line). The main resistive contributions R_2_ and R_3_ slowly grow during long-term cycling. However, the impact of this change is minimal, and aligned with the observed slight capacity loss during cycling. We suspect that both are connected to the temperature changes in the glovebox over the course of the long-term experiment. Importantly, for a 1 µm LiPON film with room-temperature ionic conductivity of ~2.5 × 10^−6^ S cm^−1^, the electrolyte area-specific resistance is on the order of 40 Ω cm^2^ (Figure 3(b,c)), which gives an ohmic overpotential of ~0.6 V at 12 mA cm^−2^. This agrees well with the observed overpotential in Figure 2(a,b), suggesting that the limiting factor in the cells presented here is the solid-state electrolyte. Therefore, the C_60_ anode could perform even at higher current densities if a lower area-specific resistance solid-state electrolyte was successfully implemented (through either a more conductive or thinner layer).

## Conclusions

This work establishes four key aspects of the C_60_ films: (1) the active layer is structurally disordered in the as-deposited state (Figure 1(c,d)); (2) the electrochemical response is faradaic and multistep (Figure 2(a,b)), providing up to 560 mAh/g; (3) the film retains substantial reversible capacity over a wide current-density range with ~70% capacity retention at 75 C and ~40% at 1600 C (Figure 2); (4) long-term cycling is very stable for thousands of cycles with negligible capacity fade and impedance growth after > 7000 cycles (Figure 3(a-c)). The evaporated C_60_ films serve as vacuum-processable, Li metal-free host anode with exceptional current-density tolerance and cycling stability in LiPON-based TFSSBs.

## Methods

### Sample preparation

Boro-Aluminosilicate glass (CB-0111, Delta Technologies, 25 × 25×1.1 mm) was cleaned and coated with 200 nm of Cu (5N, Thermo Fischer Scientific) as a current collector using evaporation in a Nextdep thermal evaporator (Angstrom Engineering, Canada). A 200 nm C60 layer was then evaporated in a separate Nextdep thermal evaporator. Another set of samples with 20 nm thickness was made for the EIS comparisons done in Figure 3(b). Fullerene C60 (99.5%, Xi’an Yuri Solar Co.) was placed in a resistively heated crucible and deposited under high vacuum (≈10^−7^ mbar). The deposition rate was controlled at 0.2 Å/s using a quartz crystal microbalance (QCM), ensuring precise thickness control and uniform film growth. The crucible temperature increased from approximately 400°C at the beginning of the evaporation process to about 520°C at the end of the deposition over the 167 minutes of the deposition. This is to maintain the deposition rate during the process. The substrates were continuously rotated during deposition to improve thickness homogeneity.

Custom shadow masks were used to pattern the depositions. For the C60 layer, a mask of an array of circles (5 mm^2^) was used. Then, 1 µm of LiPON solid state electrolyte was sputtered through a mask of an array of teardrop shapes, larger than the circles used for the C60 layer. LiPON was co-sputtered from Li_3_PO_4_ and Li_2_O targets 2’’ in diameter, in an Orion sputtering system (AJA International, USA), in confocal arrangement, at 50 sccm of N_2_ flow, at a pressure of 3 mTorr. This was then followed by a layer of Li metal (99+%, Thermo Fischer Scientific), that would act as top current collector, pseudo-reference electrode and source of Li in the cell. The 2 µm Li layer was evaporated in the Nextdep thermal evaporator (Angstrom Engineering, Canada), using a stainless steel crucible arc-coated with alumina. The Li layer deposition used a smaller teardrop shadow mask, inset from the electrolyte layer but offset from the C_60_ layer (also see Figure 1(b)). Thicknesses of layers were calibrated using profilometry and cross-sectional scanning electron microscopy (SEM) measurements.

### Electrochemical characterisation

All electrochemical tests were performed in an Ar-filled glovebox, at room temperature, without using any encapsulation. Squidstat potentiostats (Admiral Instruments, USA) were used. Squidstat Plus for any experiments involving electrochemical impedance spectroscopy (EIS) and Squidstat Prime for all other experiments. Gravimetric capacity and current density values were calculated assuming 200 nm of thickness (confirmed by profilometry), 5 mm^2^ area (as defined by the shadow masks) and assuming a density of 1.65 g/cm^3^ [39]. All C-rates stated in the manuscript are real C-rates (1/discharge time).

EIS was performed between 2 MHz and 10 mHz, with 12 steps per decade, after charging to 3 V and relaxing to OCV. The excitation amplitude was set to 50 mV. The spectra were fitted using Relaxis (rhd instruments GmbH & Co. KG, Germany).

### Other characterisation

X-ray diffraction experiments were performed using ANanalytical X’Pert PRO system (Malvern Panalytical, UK) (Cu-Kα_1_ radiation, 1.5406 Å) and a X’Celerator linear detector operating in Bragg-Brentano geometry (θ/2θ) in the range of 2θ from 10° to 70°.

SEM characterisation and TEM lamella preparation were done using a FEI Helios 660 NanoLab dual beam focused ion beam scanning electron microscope (FIB-SEM, USA). Generally, a 2 kV electron beam with an Everhard-Thornley secondary electron detector was used. For the TEM lamella preparation, Ga ion beam at 30 kV was used for initial preparation, following a standard procedure. Progressively decreasing beam currents and accelerating voltages were used for lamella thinning (down to 5 kV and 40 pA). The prepared lamellae were transferred, through an Ar-filled glovebox to a TEM using an airless transfer holder (Gatan, USA). TEM experiments were performed on a Jeol JEM-F200 microscope operated at 200 kV accelerating voltage (Japan).
